# Predicting hydrocarbon presence in marine cold seep sediments using machine learning models trained with benthic bacterial 16S rRNA taxonomy

**DOI:** 10.1128/spectrum.03033-24

**Published:** 2025-08-20

**Authors:** Rohan Khan, Tulika Bhardwaj, Carmen Li, Anirban Chakraborty, José M. Seoane, James M. Brooks, Bernie B. Bernard, Adam MacDonald, Natasha MacAdam, Calvin Campbell, Martin Fowler, Casey R. J. Hubert

**Affiliations:** 1 Geomicrobiology Group, Department of Biological Sciences, University of Calgary 98634, Calgary, Alberta, Canada; 2 Department of Biological Sciences, Idaho State University 166927, Pocatello, Idaho, USA; 3 Repsol SA 16680 , Madrid, Spain; 4 TDI-Brooks International, College Station, Texas, USA; 5 Natural Resources Canada, Geological Survey of Canada Atlantic 186595, Dartmouth, Canada; 6 Nova Scotia Department of Natural Resources and Renewables, Government of Nova Scotia 113430, Halifax, Canada; 7 Applied Petroleum Technology Canada, Calgary, Alberta, Canada; Connecticut Agricultural Experiment Station, New Haven, Connecticut, USA

**Keywords:** hydrocarbons, environmental microbiology, machine learning, 16S rRNA, marine cold seeps

## Abstract

Hydrocarbon seepage in marine sediments exerts selective pressure on benthic microbiomes. Accordingly, microbial community composition in these sediments can reflect the presence of hydrocarbons, with specific groups being more prolific in association with seepage. Here, we tested machine learning models with large 16S rRNA gene amplicon data sets derived from marine sediments in deep-sea hydrocarbon prospective areas of the Eastern Gulf of Mexico and NW Atlantic Scotian Slope. Utilizing H2O’s AutoML machine learning platform, it was determined that Gradient Boosting Machines performed best for creating 16S rRNA-based models that successfully predict the presence of hydrocarbons. Feature importance scores from the models revealed that in Gulf of Mexico samples, members of the *Aminicenantia* class (within the *Acidobacteriota* phylum) and the *Sulfurovum* genus (within the *Campylobacterota* phylum) were most diagnostic for the presence of low molecular weight hydrocarbon gases. The *Campylobacterota* lineage was also important in Scotian Slope sediments, along with sequences affiliated with the class-level JS1 group (within the *Caldatribacteriota* phylum) for determining hydrocarbon-positive sites. Testing these models in geographically distant seafloor basins showed that the microbial communities between basins varied sufficiently to prevent consistently accurate reciprocal predictions. However, models trained on a combined data set and filtered for important features performed substantially better, supporting the feasibility of generalized models under stringent feature selection. These results highlight the potential of seabed microbial taxonomy-based hydrocarbon seep site prediction when paired with refined sampling and consistent geochemical characterization.

**IMPORTANCE:**

Our study showcases an important use of bioinformatics in an interdisciplinary context, by combining hydrocarbon geochemistry and microbial biodiversity DNA sequencing profiles. We trained and compared different machine learning models on 16S rRNA-based bacterial taxonomy data using 377 DNA sequencing libraries from marine surface sediments in two different hydrocarbon prospective marine basins from different parts of the global ocean to predict the hydrocarbon status of sediment samples. Of all algorithms tested, Gradient Boosting Machines worked best for this objective. Feature importance scores from the models highlighted that in Gulf of Mexico samples, members of the *Aminicenantales* order and *Campylobacterota* lineages were most diagnostic for the presence of low molecular weight hydrocarbon gases. The *Campylobacterota* lineage was also important in NW Atlantic Scotian Slope sediments, along with sequences affiliated with the class-level group JS1 (within the *Caldatribacteriota* phylum) for determining hydrocarbon-positive sites, though several features appeared to be basin-specific. Importantly, models had a high prediction accuracy when predicting samples from the same basin but were less effective in predicting the hydrocarbon status in reciprocal basin testing, pointing to the ecological differences in hydrocarbon-driven environmental selection in different parts of the ocean. However, combined models using a refined set of predictive features improved cross-basin performance, highlighting the feasibility of a broader application. Results highlight the exciting potential of microbial taxonomy-based machine learning models in predicting broader ecological, oceanographic, and geological phenomena at the biosphere-geosphere interface.

## INTRODUCTION

Deep-sea sediments where water depths generally exceed 1,000 meters are predominantly oligotrophic environments with minimal production or influx of organic matter. Despite these conditions, diverse microbial ecosystems can persist in the seabed in the presence of oxic or anoxic conditions, varying chemical gradients, and permanently cold temperatures (1). Geological features such as cold seeps and hydrothermal vents punctuate the oligotrophic seabed and supply additional nutrients, thereby becoming hotspots of biodiversity (2). These features can expel hydrocarbon gases like methane and liquid thermogenic hydrocarbons originating in deep subseafloor environments (3). At cold seeps, the release of hydrocarbons up through the sediment to the ocean floor can select for microbial populations that take advantage of gas and oil as substrates or that are otherwise associated with thermogenic hydrocarbons. Distinct environmental niches that result from geological features influence the resident microbiome, ultimately resulting in a unique biodiversity signature compared to the surrounding seabed, which lacks an advective influx of energy-rich substrates (4).

The Eastern Gulf of Mexico and Scotian Slope in the Northwest Atlantic Ocean are both petroleum basins featuring hydrocarbon seepage. In recent studies, microbial diversity assessments using 16S rRNA gene amplicon sequencing alongside various hydrocarbon geochemistry parameters have been used to correlate certain bacterial taxa with hydrocarbon-positive sediments in these basins (5, 6). Statistical tools identified taxa belonging to lineages within the phyla *Caldatribacteriota*, *Campylobacterota,* and *Acidobacteriota* in surface sediments as being significantly associated with hydrocarbons in these and other areas (5–8).

Although prior studies of seep sediments have identified relationships between microbial lineages and the presence of different hydrocarbons, they do not take into account the microbiome in its entirety. A given marine sediment sample cannot confidently be identified as hydrocarbon-positive solely based on the existence of a small set of indicator lineages such as the three phyla cited above. Rather, the entire microbial community should be comprehensively assessed for making such a prediction (9). Machine learning algorithms can potentially achieve these objectives. Although these algorithms operate similarly to other statistical tools, they differ in scope by deriving inferences and correlations from input data (9–11). Instead of only identifying certain taxa as being correlated to hydrocarbons, machine learning models can be trained to rank all taxa present in an input data set from sediments of known hydrocarbon status and subsequently use these rankings to predict the hydrocarbon status of a new data set (9, 12). Various machine learning algorithms differ in terms of use cases and the size of data sets that can be accommodated. For predicting whether a marine sediment sample is hydrocarbon-positive or negative, binomial algorithms such as Support Vector Machines (SVM) or Gradient Boosting Machines (GBM) can be used (11, 13). These models can consider the relative abundance of each taxon within a 16S rRNA gene library, along with its taxonomic classification, and a binomial input classifying a sample’s hydrocarbon status. Generated models can then predict the hydrocarbon status of new samples based on amplicon library profiles by comparing the relative abundance of different taxa with the training data set (14, 15).

In this study, amplicon libraries from earlier studies of marine sediments in the Eastern Gulf of Mexico and Scotian Slope basins were considered in the context of the hydrocarbon status of each sample to train different machine learning algorithms and compare their performance. The goal of this approach is the creation of predictive models that can accurately classify seabed samples as hydrocarbon-positive or negative based on bacterial community structure. Providing these data sets as input for machine learning algorithms enabled determinations of the most suitable algorithms while also identifying taxonomic patterns indicative of hydrocarbons in marine surface sediments.

## MATERIALS AND METHODS

### Sampling marine sediments

**Fig 1:**
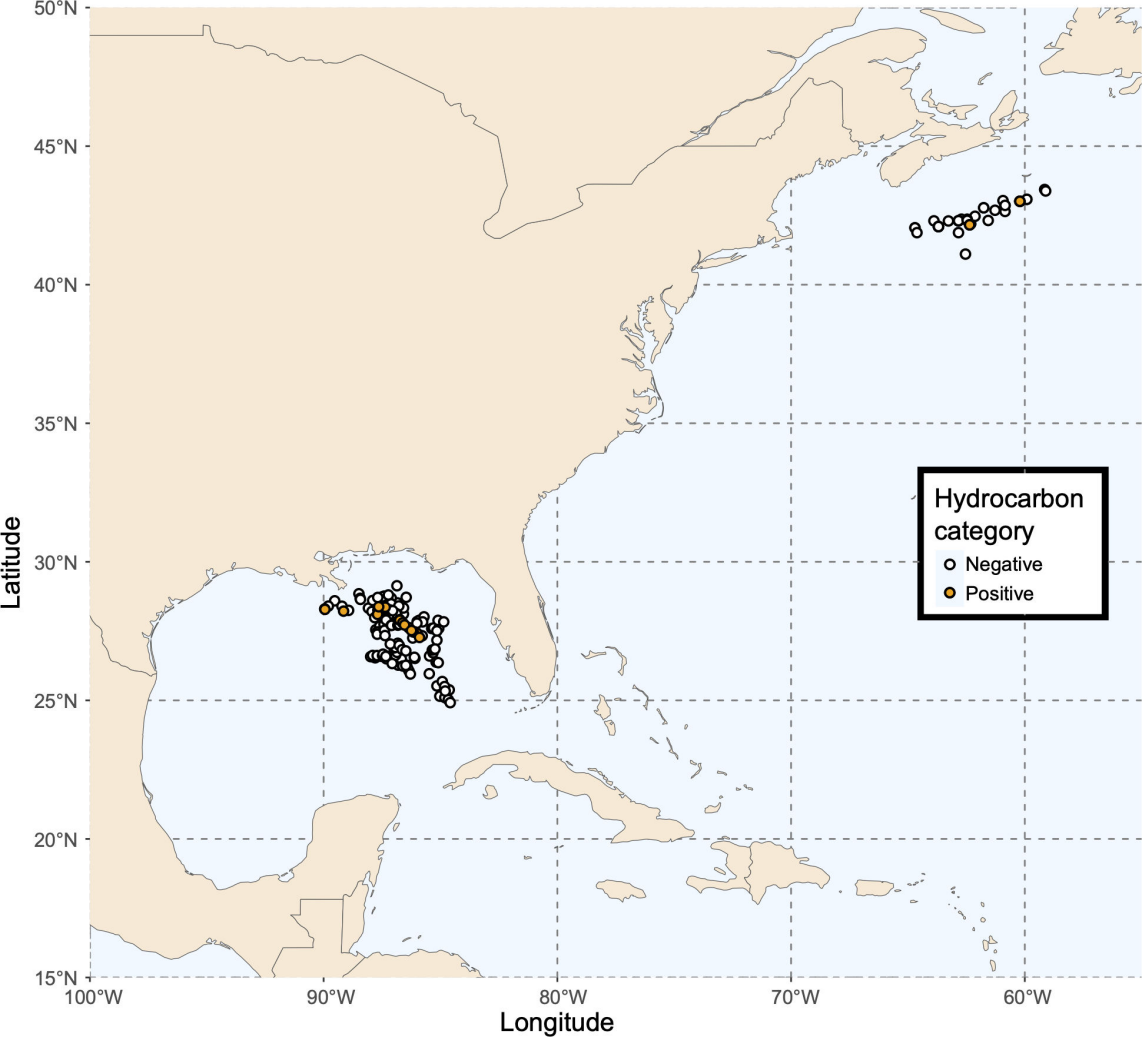
Marine sediment sampling locations in the Eastern Gulf of Mexico and Scotian Slope. Sites have been classified as hydrocarbon-positive or -negative based on geochemical testing. Gulf of Mexico samples were taken from the top 0–20 cm layer of sediment cores, while Scotian Slope sediments were sub-sampled from various depths in the cores, from 0 cm to 600 cm. The map in Fig. 1 was generated using the R package rnaturalearth, which retrieves geographic data from Natural Earth (https://www.naturalearthdata.com/).

Marine sediments from the Eastern Gulf of Mexico (Fig. 1) were sampled from January to March of 2011 aboard the RV GeoExplorer, as part of the Surface Geochemical Exploration program of TDI-Brooks International (5). Sediment samples were obtained using piston cores, which extracted sediment from 2.2 m to 5.8 m below the seafloor. Once extracted, cores were split into 20 cm sections. Surface sediment (0–20 cm) was used for DNA sequencing, while sediment from the bottom of the core was used for hydrocarbon gas analysis. Surface sediment samples, once extruded, were placed in sterile Whirl-Pak bags and frozen at −20°C, and samples from the bottom of the core were placed in metal canisters with N_2_ in headspace. In both cases, minimal air exposure was ensured (5, 16). In total, 172 piston cores were taken from 0 to 20 cm, resulting in 172 surface marine sediment samples that were each assessed by 16S rRNA gene sequencing (5).

Marine sediments from the Scotian Slope in the NW Atlantic (Fig. 1) were obtained in 2015, 2016, and 2018 aboard the CCGS Hudson (6, 17). In 2015 and 2016, sediment samples were obtained using piston cores, which were up to 10 m in length. In 2018, sampling was conducted with gravity cores up to 6 m in length (6, 18). The cores were sectioned into 1.5 m segments and inspected for signs of hydrocarbon seepage. Sediment samples from different depths were stored at −20°C in aluminum foil for eventual organic matter extractions, and the bottom few centimeters of every core were sampled into an Isojar for eventual hydrocarbon gas analysis. For all sampled depths, roughly 2 mL of sediment was sub-sampled and frozen at −80°C aboard the vessel for eventual DNA extraction. In total, 54 different locations were sampled (Fig. 1) at various depths such that 205 different sediment sub-samples were used for 16S rRNA gene sequencing (6).

### Hydrocarbon characterization of marine sediments

Concentrations of hydrocarbon gases associated with Eastern Gulf of Mexico samples were measured using sediment sampled from the bottom of piston cores that were stored in gas canisters upon sampling on board the ship (5). The canisters were placed at 40°C for 4 hours, with the lid of the canister being silicone glued to silicone septa. Using a high-speed shaker, the light hydrocarbons dissolved in interstitial water were equilibrated with the nitrogen gas in the canister headspace (5). Hydrocarbon gases were subsequently quantified using gas chromatography (GC) by TDI Brooks International (College Station, USA). Based on industry-relevant concentration thresholds of hydrocarbon gases, samples were classified as gas-positive or gas-negative.

A different methodology was used to assess the presence of thermogenic hydrocarbons in Scotian Slope sediments. The analyses were performed by Applied Petroleum Technology (Oslo, Norway) as described in Li et al. (6) and summarized below. Sediment from the base of each core was collected in an Isojar for headspace gas analysis, with additional samples collected if there were obvious signs of gas in other parts of the core. GC of the headspace gas was used to determine concentration and C_1+_ composition, with isotope analysis performed on samples with sufficient volume. Additional sediment samples throughout the cores were collected and frozen for eventual total organic carbon (TOC) analysis (based on appearance and lithology). Extractions used a Tecator Soxtec system with the extractable organic matter (EOM) analyzed using GC. Based on the EOM gas chromatograms and the headspace gas results, about a third of the samples were selected for more detailed analyses involving fractionation of the EOM and gas chromatography-mass spectrometry (GC-MS) of the hydrocarbon fractions.

Geochemical data were used to rank each sediment coring location according to its hydrocarbon status. Eastern Gulf of Mexico samples were broadly classified as being gas-positive or gas-negative based on GC results (5, 19). Scotian Slope samples were classified based on headspace gas analysis and EOM results and were given overall rankings of thermogenic, biogenic, negative, or inconclusive. For the training of machine learning models in this study, inconclusive samples were excluded as a means of aiding in model interpretability through noise and variance reduction. To facilitate using binomial models, the biogenic and thermogenic samples were reclassified together into a single “hydrocarbon-positive” category, thereby streamlining comparisons based on the presence of hydrocarbons in samples from both the Scotian Slope and Eastern Gulf of Mexico data sets.

### DNA extraction and sequencing

Approximately 0.5–1.0 g of sediment was used for genomic DNA extraction employing the DNeasy PowerLyzer PowerSoil kit (Qiagen) following a version of the provided protocol. The v3-v4 region of the 16S rRNA gene was PCR amplified from the extracted DNA using bacterial 16S rRNA gene primers SD-Bact-0341-bS17/SD-Bact-0785-aA21 primers (5, 6, 20). After PCR, the resulting amplicon libraries were barcoded and prepared for sequencing using a slightly modified version of Illumina’s 16S Metagenomic Sequencing Library Preparation protocol (21). Libraries were sequenced using an Illumina MiSeq benchtop sequencer with a 600 cycle MiSeq Reagent V3 kit.

### Phylogenetic analysis

For Eastern Gulf of Mexico samples, 172 different surface sediment (0–20 cm) DNA sequencing libraries were prepared and were available for training machine learning models. For the Scotian Slope, DNA extractions were conducted in triplicate, resulting in 549 libraries that could be used as input to train machine learning models. Once amplicon sequencing libraries were generated, primer sequences were removed using Cutadapt (22), and primer-free raw reads were processed using the R package DADA2 (23). Forward reads were truncated to 250 nucleotides, while reverse reads were truncated to 220 nucleotides. Reads were removed if the expected error was greater than 2. Sequence-specific errors were removed, and the forward and reverse reads were merged with the removal of chimeras. Amplicon sequence variant (ASV) tables were constructed separately for Scotian Slope and Eastern Gulf of Mexico samples. Subsequently, taxonomy was assigned using the SILVA database version 138 (24). Unclassified ASVs, at any taxonomic level, were renamed to the closest identifiable taxonomic level. For example, if an ASV was classified as “*Firmicutes*” at the phylum level using the SILVA database, but was unclassified at the class level, the class was labeled as “*Firmicutes*_Phylum.” To limit potential overfitting of machine learning models due to technical replicates being available for some samples and not others, a single representative ASV abundance profile was created for each location and sediment depth by combining abundance data from all available replicates. This approach reduces model bias toward certain sites during the training process. To achieve this, raw ASV read data were combined to determine average values across replicates. In total, there are 172 Eastern Gulf of Mexico samples (11 gas-positive and 161-negative) and 205 Scotian Slope samples (31 hydrocarbon-positive and 174 hydrocarbon-negative).

### Utilizing machine learning to identify relative feature importance of taxonomic clades for predicting sediment hydrocarbon status

The H2O R package, cluster version 3.40.0.4, was used to perform machine learning applications, specifically using the AutoML function, with Distributed Random Forest (DRF), Extremely Randomized Trees (XRT), Generalized Linear Model (GLM), XGBoost, multiple H2O Gradient Boosting Machines (GBM), DeepLearning, and StackedEnsemble algorithms (25). ASV abundance tables for either the Eastern Gulf of Mexico samples (*n* = 172) or the Scotian Slope samples (*n* = 205) were prepared, containing the number of reads for each ASV and associated taxonomic classifications. The data were combined with the hydrocarbon status of the site that each sample was derived from. For the algorithms, taxonomic clade names for each ASV, specific to a taxonomic level, served as the features for training. Upon training with a data set, the feature importance of the resulting models can be observed. This reveals which taxonomic clades, relative to others, play the most important role in predicting whether a site is hydrocarbon-positive or negative.

ASVs with less than 10 reads in total spanning all libraries in a specific data set were removed to simplify the data set and avoid unnecessary overfitting by machine learning models. The data were then normalized by dividing ASV reads by the total number of reads per sample. Models were constructed based on the relative number of reads from the entire data set associated with each sample. Individual models were tailored to different taxonomic levels, utilizing only the ASV IDs at that particular level as the identifying features.

For training machine learning algorithms, the hydrocarbon status of a sample was used as the target variable, and taxonomic names were used as the features to train models. The entire data set for the respective basin was used for training the models. AutoML was used, excluding the “DeepLearning” and “StackedEnsemble” algorithms. To address category imbalance (commonly referred to as *class* imbalance, but here termed *category* to avoid confusion with taxonomic class) given that both basins have more hydrocarbon-negative samples than hydrocarbon-positive samples, the Synthetic Minority Over-sampling Technique (SMOTE) was applied using the smotefamily R package. SMOTE generates synthetic replicates of the minority category (hydrocarbon-positive samples) through interpolation of each hydrocarbon-positive sample and its K nearest neighbors (K = 5; dup_size = 3) (26, 27). This increases the number of minority category samples without duplicating any existing hydrocarbon-positive samples. Final models were generated using stratified 10-fold cross-validation, which involved splitting the training data set into 10 random folds, then utilizing 9 of the folds for training and one for testing. Each iteration utilized a set of 9 folds for training and a unique test set. Models generated from each iteration were evaluated using validation metrics determined by H2O, and a final model was generated taking into account metrics from the 10 iterative models. Cross-validation was conducted to generate models for each type of algorithm (25). Algorithms were then sorted based on Area Under the Precision-Recall Curve (AUCPR), which is a performance metric suited for imbalanced categories (28, 29). The model with the highest AUCPR score was chosen. AUCPR scores range between 0 and 1 such that a score of 1 indicates perfect recall (i.e., the proportion of hydrocarbon-positive samples that were correctly predicted to be hydrocarbon-positive) and precision (i.e., the proportion of hydrocarbon-positive predictions that were correct) while a score of 0.5, in a balanced category model, indicates that the model is no better at prediction than randomly guessing (30). In addition to AUCPR, the top model’s F1 score was determined to evaluate model performance. F1 score is a harmonic mean of precision and recall, where a score closer to 1 indicates a model’s ability to balance between precision and recall. Both F1 and AUCPR are useful metrics for indicating the performance of models generated using an imbalanced category (29, 31). Here, both metrics are derived from cross-validation results using the h20.performance() function with xval = TRUE. Feature importance was identified for the algorithm with the largest AUCPR score. This workflow was performed for both the Eastern Gulf of Mexico and Scotian Slope basins, at phylum-, class-, order-, family-, and genus-level taxonomies.

### Using machine learning models generated using one basin to predict the hydrocarbon status of another basin

To determine whether models trained on the Eastern Gulf of Mexico data set can accurately predict the hydrocarbon status of the Scotian Slope samples, GBM models previously trained with the Gulf of Mexico data set and with the highest AUCPR scores at each taxonomic level were used. Scotian Slope data were then tested using this model without the associated hydrocarbon status. The model then provides a classification score for each Scotian Slope sample to indicate the likelihood of a sample being hydrocarbon-negative. Samples with classification scores above a threshold were predicted to be hydrocarbon-negative, and those below were predicted to be hydrocarbon-positive. This threshold was determined using the cross-validation training results of the respective model, specifically the value maximizing the F1 score. To evaluate the performance of the reciprocal testing, precision, recall, and F1 scores were calculated based on the prediction outcomes. The reciprocal test was also performed for the Eastern Gulf of Mexico data set, with previously generated Scotian Slope GBM models used for prediction.

### Using models generated with a combined data set of amplicons from both basins

To explore the similarities between hydrocarbon indicator species across different basins and the effectiveness of a model trained on relative abundances from different locations, combined training and testing data sets were generated. All hydrocarbon-positive and gas-positive samples (42 in total) were combined and randomly split into two halves using a fixed seed. Similarly, all hydrocarbon-negative samples (336 in total) were combined and randomly split into two halves. The data used for training models (the training set) consisted of half of the hydrocarbon-positive samples and half of the hydrocarbon-negative samples. The second halves of the samples were combined to make an external testing data set (the test set). This training data set contains Eastern Gulf of Mexico and Scotian Slope amplicon libraries, with classifications at each taxonomic level, and the respective hydrocarbon status of each sample (i.e., positive or negative). Both the training and test sets were further refined to only include features (i.e., taxonomic clades) that were determined to have a relative feature importance of greater than 0.1 in either of the two previous top-performing basin-specific. Remaining features were removed as a means of reducing data complexity and overfitting, such that trained models could be better generalized. This filtering was performed for each taxonomic level before train-test splitting. GBM algorithms were trained on the trimmed training data set at five different taxonomic levels (phylum to genus) after using SMOTE, as previously described, for category balancing.

Final models were then generated using 10-fold cross-validation with the refined training data set, and the models with the largest AUCPR scores were chosen. To determine the effectiveness of the models, the testing data set was provided to the generated models without the hydrocarbon status of the different samples. Similar to the reciprocal testing, each sample from the testing set was assigned a classification score to indicate how likely the sample is to be hydrocarbon-negative. The classification threshold was determined using cross-validation data. Precision, recall, and F1 score were calculated to evaluate model performance based on the predicted hydrocarbon status compared to the measured status of the test set. To account for any sampling bias from randomizing the training and testing data sets, this process was repeated two more times using different random number generators (seeds).

## RESULTS

### Feature importance of taxonomic clades in Eastern Gulf of Mexico sediments

**Fig 2:**
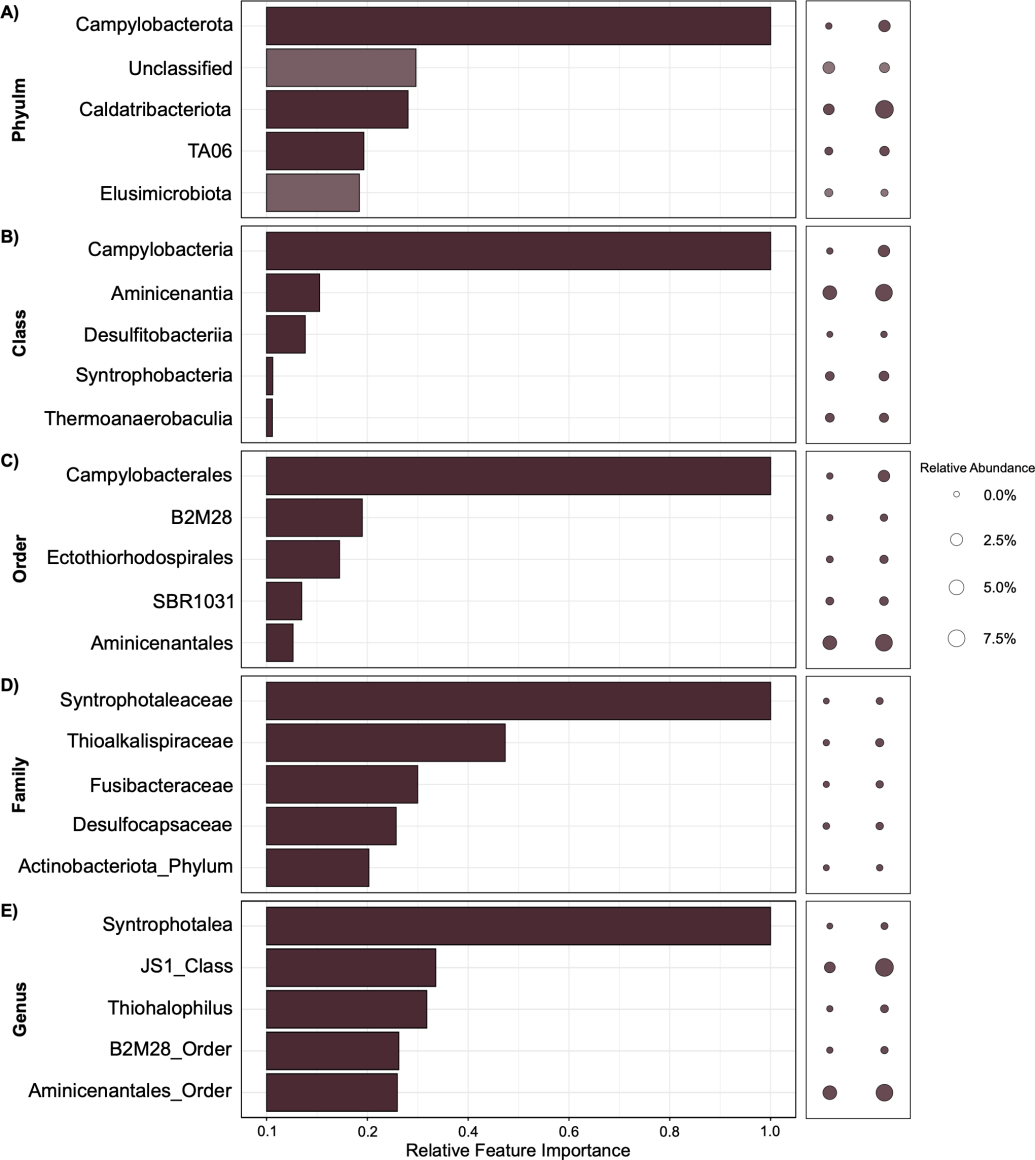
Eastern Gulf of Mexico amplicon libraries from 11 gas-positive and 161 gas-negative samples were assessed using different machine learning algorithms. Bar plots show results from H2O’s Gradient Boosting Machine (GBM) algorithm, which had the highest AUCPR score, compared to all other AutoML algorithms. The feature importance of the top five diagnostic taxa at phylum (A), class (B), order (C), family (D), and genus (E) levels is shown, indicating their relative importance in predicting whether a sediment sample is gas-positive or gas-negative. Models were generated using aggregate abundances for different taxonomic groups combined with hydrocarbon gas metadata associated with each sample as input features. Bubble plots show relative abundances of each taxon collectively in all 161 gas-negative samples (left; 4,318,775 total reads) and all 11 gas-positive (right; 727,639 total reads). Model-specific F1 and AUCPR scores are reported next to the respective taxonomic level. The taxa in higher relative abundance in gas-positive samples are highlighted by a darker shade, whereas lighter bars correspond to taxa that have indicator potential based on not being observed in gas-positive samples.

H2O Gradient Boosting Machine (GBM) algorithms provided trained models with the highest AUCPR scores. These were 1.0000 at phylum, class, and order levels, 0.9990 at the family level, and 0.9995 at the genus level (Fig. 2). Corresponding F1 scores—based on cross-validation—were similar, with phylum, class, and order all being 1.0000, and family and genus levels 0.9885 and 0.9888, respectively (Fig. 2).

The entire Eastern Gulf of Mexico data set of 172 bacterial amplicon libraries was used to train models at phylum, class, order, family, and genus levels, together with the hydrocarbon status of each sample. Feature importance plots identified *Campylobacterota* as being a strong phylum-level predictor of hydrocarbon gas status in the Eastern Gulf of Mexico sediments (Fig. 2A). Sites that have hydrocarbon gases present also exhibited a higher relative abundance of *Campylobacterota* (2.09%) compared to gas-negative samples (0.04%) (Fig. 2A). Conversely, *Elusimicrobiota* was also within the top five phylum-level features, albeit at lower importance relative to *Campylobacterota* owing to higher relative abundance in gas-negative samples (Fig. 2A; Table S1). These were the only features greater in gas-negative relative abundance across all taxonomic levels identified as being within the top five features of the Eastern Gulf of Mexico models. *Caldatribacteriota* was also present as an important phylum, being greater in relative abundance in gas-positive samples (9.01%) than in gas-negative samples (1.63%).

Different lineages were highlighted at the class level, where *Campylobacteria* was identified as being the most important class-level lineage by the GBM model while also being in greater relative abundance in gas-positive samples. The class *Aminicenantia* (belonging to the phylum *Acidobacteriota*) was also identified as an important feature while also being detected in greater relative abundance in gas-positive samples (7.54% as compared to 4.03%) (Fig. 2B; Table S1). At the order level, *Camplyobacterales* is most important for successfully predicting hydrocarbon status using the GBM model (Fig. 2C). Although notably less important, *Aminicenantales* (*Acidobacteriota* phylum) was also within the top five features at the order level. *Syntrophotaleaceae* at the family level and its genus *Syntrophotalea* are the best predictors at finer levels of taxonomic resolution, despite being in very low relative abundance in both gas-positive (0.11%) and gas-negative samples (0.003%) (Fig. 2D and E). At the genus level, *Aminicenantales*_Order and JS1_Class are two of the top five genera; however, their relative importance is lower than *Syntrophotalea* (Fig. 2E).

It should be noted that certain ASVs, such as those part of the *Aminicenantales* order, could not be classified at more refined taxonomic levels using the SILVA database (version 138). Therefore, in such cases, the order name was carried over and applied for training models at these finer-scale taxonomic levels.

### Feature importance of taxonomic clades in NW Atlantic Scotian Slope sediments

**Fig 3:**
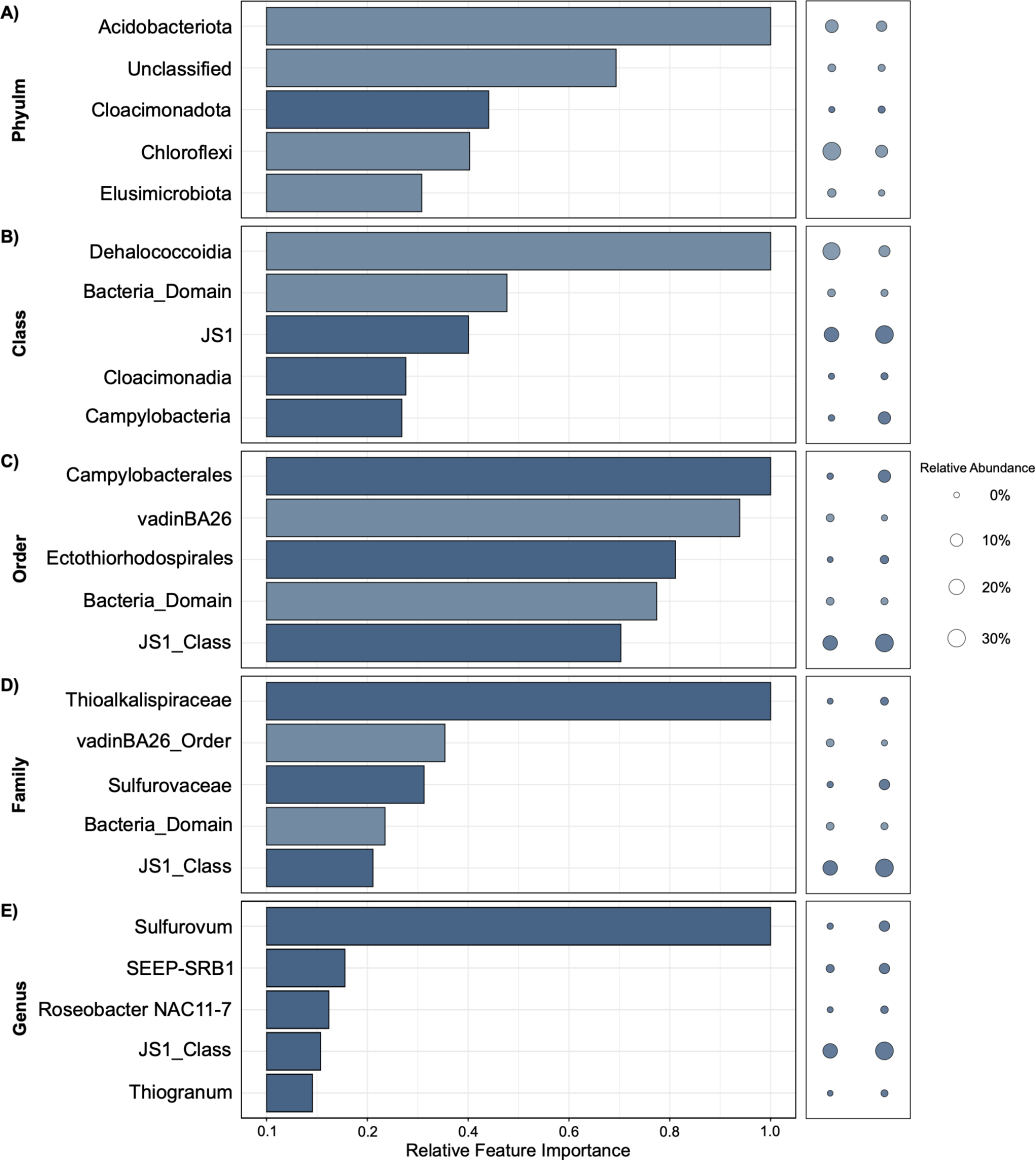
Scotian Slope amplicon libraries from 31 hydrocarbon-positive and 174 hydrocarbon-negative samples were assessed using different machine learning algorithms. Bar plots show results from H2O’s Gradient Boosting Machine (GBM) algorithm, which had the highest AUCPR score, compared to all other AutoML algorithms. The feature importance of the top five diagnostic taxa at phylum (A), class (B), order (C), family (D), and genus (E) levels is shown, indicating their relative importance in predicting whether a sediment sample is hydrocarbon-positive or hydrocarbon-negative. Models were generated using aggregate abundances for different taxonomic groups combined with hydrocarbon metadata associated with each sample as input features. Bubble plots show relative abundances of each taxon collectively in all 174 hydrocarbon-negative samples (left; 10,713,491 total reads) and all 31 hydrocarbon-positive (right; 1,718,568 total reads). Model-specific F1 and AUCPR scores are reported next to the respective taxonomic level. The taxa in higher relative abundance in gas-positive samples are highlighted by a darker shade, whereas lighter bars correspond to taxa that have indicator potential based on not being observed in hydrocarbon-positive samples.

GBM models also consistently resulted in the largest AUCPR scores for the Scotian Slope data set, reaching 1.0000 or 0.9999 at all taxonomic levels (Fig. 3). Similarly, F1 scores based on cross-validation were also high at either 1.0000 or 0.9960. In Scotian Slope sediments, *Acidobacteriota* emerged as the top phyla for predicting the presence of hydrocarbons (Fig. 3A). At the phylum level, *Acidobacteriota* was greater in relative abundance in hydrocarbon-negative sites (11.08% compared to 4.86%) (Fig. 3A; Table S2). *Chloroflexi* and *Cloacimonadota* were also determined to be important features at the phylum level, with the former being in greater abundance in hydrocarbon-negative samples (31.81% in negative and 8.97% in positive) and the latter being in greater abundance in hydrocarbon-positive samples (0.45% in positive and 0.05% in negative) (Fig. 3A; Table S2).

*Dehalococcoidia* stands out in feature importance at the class level in Scotian Slope sediments, being greater in relative abundance in hydrocarbon-negative samples (Fig. 3B). *Campylobacteria* and JS1 are also in the top five features at the class level, but with much lower feature importance scores than *Dehalococcoidia* despite their prevalence in hydrocarbon-positive samples. At the order level, all five features have a relative importance of greater than 0.5, with *Campylobacterales* being the most important in predicting hydrocarbon status (Fig. 2C). The order-level grouping JS1_Class and *Ectothiorhodospirales*, which are greater in relative abundance in hydrocarbon-positive samples, had high feature importance scores. At the family level, *Thioalkalispiraceae* was determined to be the most important for hydrocarbon status prediction while being greater in abundance in hydrocarbon-positive samples (1.09%) compared to negative samples (0.01%). *Sulfurovaceae* (within the order *Campylobacterales*) and JS1_class are within the top five but notably less important (Fig. 2D). *Sulfurovum* is the key indicator with a higher presence in hydrocarbon-positive samples at the genus level (Fig. 2E).

### Using models from one basin to predict the hydrocarbon status in another basin

Scotian Slope-trained GBM models with the best AUCPR scores were used in reciprocal basin testing to predict the hydrocarbon status of Eastern Gulf of Mexico samples. Models provided a classification score for the likelihood of the Eastern Gulf of Mexico samples being hydrocarbon-negative. Using the cross-validation threshold determined during initial model creation, Eastern Gulf of Mexico samples were classified as hydrocarbon-negative or hydrocarbon-positive. Interestingly, regardless of taxonomic level and the geochemistry-confirmed hydrocarbon status of Eastern Gulf of Mexico sediments, all samples were predicted to be hydrocarbon-negative. As such, although accuracy is high at 0.936 across all taxonomic levels, this outcome is caused by the category imbalance in the Eastern Gulf of Mexico data set. 

**Fig 4:**
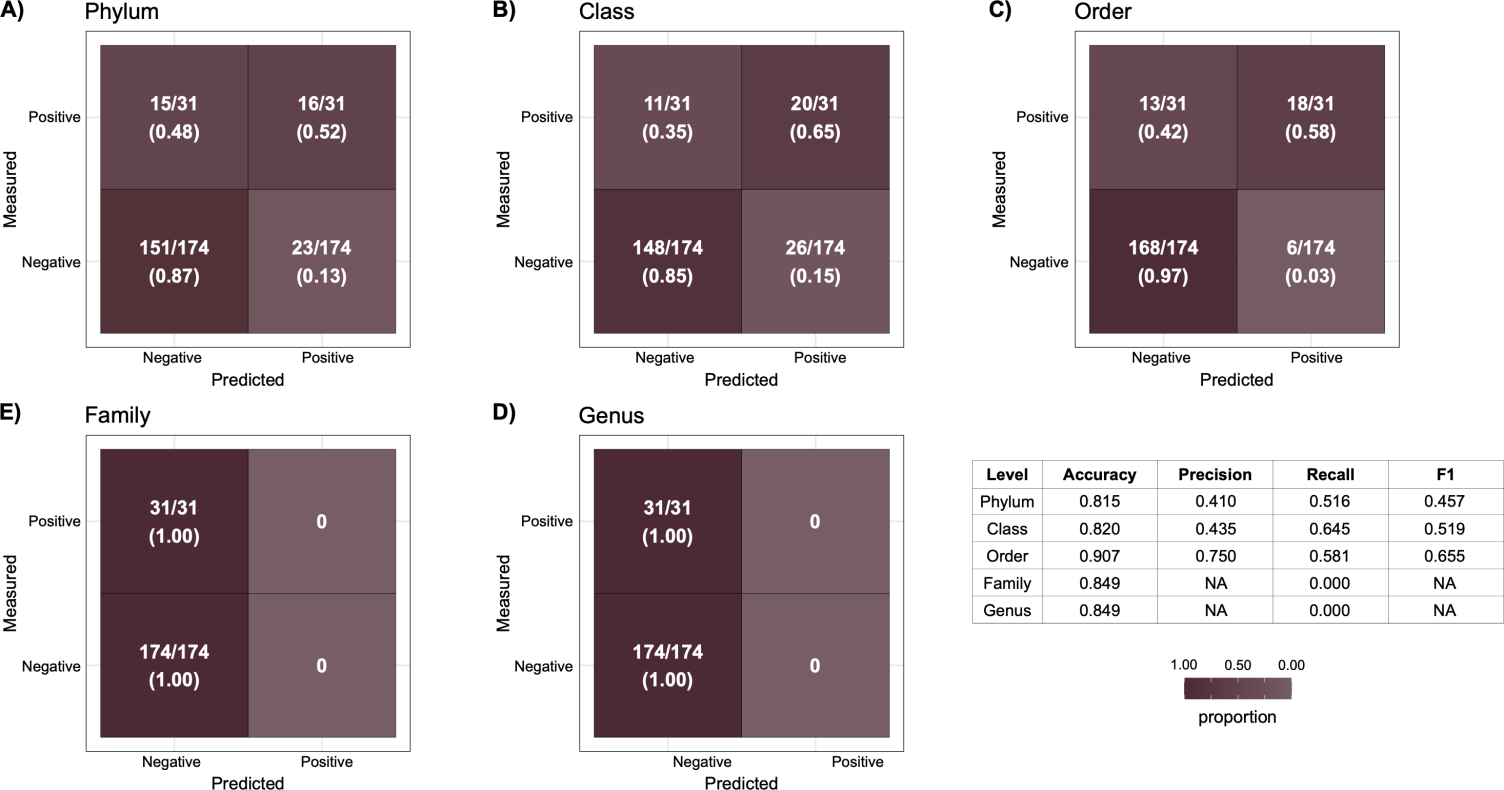
Performance of models trained on Eastern Gulf of Mexico data that were used to predict the hydrocarbon status of 31 hydrocarbon-positive and 174 hydrocarbon-negative Scotian Slope samples. For each taxonomic level, the best Eastern Gulf of Mexico model (determined by AUCPR score) was used to make predictions on the Scotian Slope test set. For each sample, a classification score indicating the likelihood of the sample being hydrocarbon-negative was obtained. Predictions used the classification score threshold determined by the cross-validation testing during initial model generation. If a classification score was greater than the threshold, the sample was predicted as hydrocarbon-negative, and if it was less than the threshold, the sample was predicted as hydrocarbon-positive. Panels A–E show confusion matrices at different taxonomic levels, with the number of Scotian Slope samples predicted in each cell of the confusion matrix. F showcases the accuracy, precision, recall, and F1 scores at each taxonomic level based on model performance.

**Fig 5:**
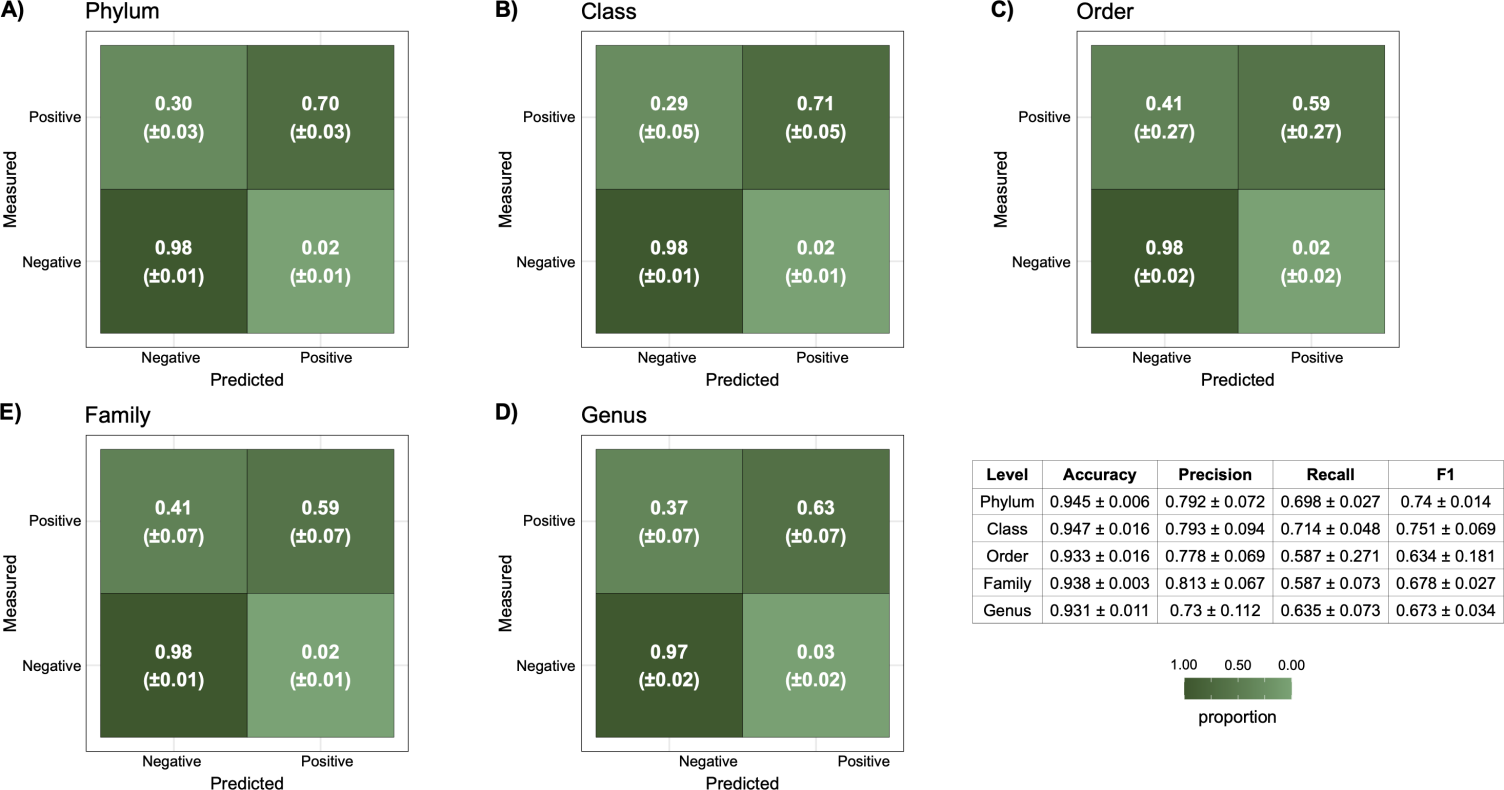
Performance of models trained on a combined half data set of Eastern Gulf of Mexico and Scotian Slope samples that were used to predict the hydrocarbon status of the other 21 hydrocarbon-positive and 168 hydrocarbon-negative samples. For each taxonomic level, the best combined model, determined by AUCPR score, was used to make predictions on the testing data set. Before training, both data sets were filtered to include only taxonomic clades with a relative feature importance of greater than 0.1 in either of the Eastern Gulf of Mexico or Scotian Slope basin-specific GBM models. For each sample, a classification score indicating the likelihood of the sample being hydrocarbon-negative was obtained. Predictions used the classification score threshold determined by the cross-validation testing during initial model generation. If a classification score was greater than the threshold, the sample was classified as hydrocarbon-negative. If a classification score was lower than the threshold, the sample was classified as hydrocarbon-positive. Randomized data set construction was repeated three times. Panels A–E show the average confusion matrix results across the replicates, with the quadrants in each plot indicating the mean and ±standard deviation. F showcases the average accuracy, precision, recall, and F1 scores at each taxonomic level across all three replicates, along with the standard deviation.

Different results were observed in the reciprocal test using Eastern Gulf of Mexico trained GBM models to predict the gas hydrocarbon status of the 205 Scotian Slope sediments (i.e., 31 hydrocarbon-positive and 174 negative). At the phylum level, 16 out of the 31 positive samples were predicted correctly, while 23 out of the 174 negative samples were predicted incorrectly (Fig. 5A). At the class level, the Gulf of Mexico model correctly predicted 30 out of 31 positive Scotian Slope samples but misclassified 26 out of 174 negative samples (Fig. 4B). At the order level, 18 out of 31 positive samples were predicted accurately, and 6 out of 174 negative samples were predicted incorrectly (Fig. 4C). Interestingly, at the family and genus levels, all samples were predicted to be hydrocarbon-negative regardless of geochemistry-confirmed hydrocarbon status (Fig. 4D and E). Overall model accuracy was high, being greater than 0.8 at all taxonomic levels (Fig. 4F). However, due to hydrocarbon-positive samples being predicted correctly at close to a rate of 50%, the overall F1 score of the phylum and class orders is close to 0.5, with the order level F1 score being a modest improvement at 0.655.

### Training models on a combined data set from both basins

Models were trained on a combined data set that included a randomly selected half of the amplicon libraries from both basins to create a training data set, with the resulting model applied to the remaining samples (the testing data set), this was repeated three times per taxonomic level to reduce sampling bias in the randomization process. To reduce overfitting, prior to model generation, both data sets were filtered to keep only taxonomic clades that had a relative feature importance of greater than 0.1 in either the top Eastern Gulf of Mexico or Scotian Slope GBM models. Compared to the reciprocal testing results, the true-positive rate (i.e., the number of samples measured to be hydrocarbon-positive that were predicted correctly) was higher across all taxonomic levels (Fig. 5A through E). In turn, the precision of the combined models is also higher, with models at all taxonomic levels having an average score greater than 0.73. Across all taxonomic levels, the F1 score, based on the predictive precision and recall metrics, was greater than 0.673 with the phylum and class level models performing best with scores of 0.74 and 0.751, respectively (Fig. 5F).

## DISCUSSION

### Bacterial associations with hydrocarbons predicted by machine learning

Earlier work on Eastern Gulf of Mexico and Scotian Slope sediments identified ASVs belonging to the phyla *Caldatribacteriota*, *Campylobacterota,* and *Acidobacteriota* as significantly associated with hydrocarbons (5, 6). Those relationships were determined using the ‘indicspecies’ test (6) or Pearson correlation coefficient (5). Machine learning applied to the same data sets, in this study, reveals more nuanced patterns as shown here using basin-specific GBM models generated with 10-fold cross-validation through H2O’s AutoML. GBM outperformed other algorithms, consistent with these data sets being tailored for binomial or binary predictions on the basis of presence or absence of hydrocarbons (13, 25). Different results arising from machine learning algorithms compared to earlier analyses of these marine sediments can be explained by the statistical methods used. The prior studies identified specific ASVs as being significantly correlated with hydrocarbon-positive sediments (5, 6). While certain ASVs might indeed be important in predicting if a site is hydrocarbon-positive, phylum-level affiliations of those ASVs might not be as diagnostic. With machine learning, all ASVs within a given taxon are considered when making predictions, not just one or a few ASVs. In addition, correlation analysis used in GBM models differs from statistical methods in the previous studies. For example, Pearson correlations in the original Eastern Gulf of Mexico analysis highlighted linear relationships, similar to how GLM models use linear or logistic regression to determine feature importance (32). GBM, on the other hand, uses a decision tree-based model, emphasizing non-linear relationships (13, 32, 33).

Both Eastern Gulf of Mexico and Scotian Slope models indicate that members of the phylum *Campylobacterota* and its affiliated lineages (i.e., *Campylobacteria*, *Campylobacterales,* and *Sulfurovum*) are important in predicting hydrocarbon-positive samples. Conversely, a more variable relationship for *Acidobacteriota* and its lineages was observed across the two basins using the GBM models. In the Eastern Gulf of Mexico models, *Acidobacteriota* lineages are sporadically identified as being important features, in greater relative abundance in gas-positive samples (Fig. 2). On the other hand, in Scotian Slope models, *Acidobacteriota* is the top phylum-level feature owing to it being more abundant in hydrocarbon-negative samples, with associated lineages not appearing within the top five features at more refined taxonomic levels (Fig. 3). Other features such as *Syntrophotaleaceae* and *Syntrophotalea* (belonging to the *Desulfobacterota* phylum) are important predictors of gas-positive sites at the family and genus levels in Eastern Gulf of Mexico sediments despite being detected in very low relative abundance. This is due to high fold changes in relative abundance between gas-positive and gas-negative samples (0.11% and 0.003%, respectively). This may mean that the biological relevance of these two taxa is less significant, raising the potential for sequencing artifacts to influence predictions that rely solely on feature importance scores determined in this way (34). Similarly, from Scotian Slope models, *Thioalkalispiraceae* (family level) and *Ectothiorhodospirales* (order level) were notably important despite low relative abundance across all samples. A more abundant and well-known seep lineage JS1 (*Caldatribacteriota* phylum) also appeared in both models, but with moderate importance and less consistency than *Campylobacterota* lineages. Overall, feature importances from these models suggest that certain taxa like *Campylobacterota* may indicate the presence of hydrocarbons across different basins (due to enhanced sulfur cycling driven by hydrocarbon inputs, that these bacteria are well known for) while the relationship between other taxa like *Caldatribacteriota* and hydrocarbon-positive sites is basin-specific (35). This may reflect some degree of ecological diversity between areas of hydrocarbon seepage in different ocean basins.

### Reciprocal basin tests indicate unique indicator species and possible overfitting

The observations above are corroborated by the results of reciprocal basin tests. Despite perfect or near-perfect AUCPR and F1 scores in the initial cross-validation, when using the model from one basin to predict the hydrocarbon status of samples from the other basin, models performed poorly. Scotian Slope models failed to identify any hydrocarbon-positive samples from the Eastern Gulf of Mexico. This indicates that the Scotian Slope models have overly conservative prediction thresholds or failed to assign hydrocarbon-negative prediction scores low enough to meet the hydrocarbon-positive cutoff. Possible overfitting to microbial feature data in the Scotian Slope data set may reflect distinctness between sediment microbiomes in the two basins, especially at hydrocarbon seeps. Eastern Gulf of Mexico models showed slightly better cross-basin performance at the phylum, family, and order levels. However, given true-positive prediction rates by this scenario being close to or less than 0.5 (i.e., no better than randomly guessing), these models are not optimal for this purpose.

The contrast between basin-specific and reciprocal-basin performance indicates that the basin-specific models are likely overfitting to their training data sets. This could be a result of two factors. First, the training data sets may have too many features (microbial taxa) that are not meaningfully contributing to the model construction and rather lead to false correlations between microbial taxa and hydrocarbon status. Second, accurate prediction is prevented by true ecological differences between microbial communities of the two basins, where certain taxa serve as hydrocarbon indicator species in one basin but not the other. While taxa like *Campylobacterota* and its sub-lineages appeared predictive in both basins, other taxa exhibited basin-specific associations. Machine learning models based on entire microbial communities may thus capture too much basin-specific “noise” to be generalizable to other basins. Broader environmental and geological factors, such as pressure, temperature, and nutrient availability, may also play a role in microbial populations being distinct in different ocean basins (36). These site-specific features can shape microbial community composition independent of hydrocarbon seepage; thus, caution should be taken when attributing community divergence solely to hydrocarbon-based selective pressures.

### Generalizability of a combined model

Upon filtering the combined data set to include only taxa with high relative feature importance from basin-specific models, F1 scores markedly improved compared to reciprocal basin testing. In a generalized combined model, across all taxonomic levels, F1 scores were greater than 0.634, reaching up to 0.740 and 0.751 for the phylum and class levels, respectively. These scores indicate a much better balance between precision and recall using a combined model. Specifically, the phylum- and class-level models performed best, likely due to lower taxonomic resolution leading to less noise being captured during model training. This allows for models to better capture broader and more conserved microbial patterns between the two basins.

Improved performance of combined models suggests that removing features with low importance reduces overfitting and enhances generalizability (37). Furthermore, the effectiveness of a combined model indicates that there are shared predictive taxa across basins that may represent broader hydrocarbon-associated microbial community patterns (38). Through continued refinement and incorporation of microbial data from additional basins beyond the two examined here, combined models may be effective as predictors of hydrocarbon seep sites using 16S rRNA gene sequencing and resulting taxonomic data.

### Model performance influenced by differences in sediment sampling and geochemical testing

Marine sediment sampling and geochemical analysis in the respective basins were not consistent, which likely contributes to the less-than-ideal reciprocal and combined test results (Fig. 4 and 5). Inconsistent hydrocarbon classification criteria, such as the reliance on gas-specific markers in the Eastern Gulf of Mexico and broader thermogenic and biogenic markers in the Scotian Slope, may have led machine learning models to learn study-specific microbial effects rather than general and ecology-driven hydrocarbon indicators (5, 6, 39, 40). Some Scotian Slope features, for example, may reflect liquid hydrocarbons associations that cannot be captured by the gas-specific criteria in the Eastern Gulf of Mexico sediment analyses. Such differences in hydrocarbon testing impact model performance and lead to training that predicts narrower definitions of hydrocarbon-positive sediment sites, reducing their ability to be generalized. Similarly, the Scotian Slope data set contains multiple amplicon libraries from the same sampling site but at different sediment depths in a given sediment core, whereas the Eastern Gulf of Mexico data set features one library per location and sediment sample. As a result, sampling redundancy is not consistent between the two. Scotian Slope-trained models may be overfit to basin-specific microbial profiles, thus high basin-specific F1 and AUCPR but reduced cross-basin performance (41, 42). Limiting both data sets to 0–20 cmbsf samples would reduce the number of unique samples, potentially leading to unreliable models due to a lack of diversity in the training data. While SMOTE helped to address category imbalance by interpolating new hydrocarbon-positive samples, synthetic samples do not reflect true biological diversity. This can lead to potential overfitting of models to specific microbial signatures already present in the hydrocarbon-positive samples, without capturing new microbial associations that may exist in seep sites that were not sampled (43). As a result, models may perform well during cross-validation but struggle with unseen microbial patterns, especially in hydrocarbon-positive sediments.

### Future directions for machine learning and microbial data

Ultimately, the issues raised above underscore the importance of harmonizing sampling and classification methodologies across study sites and implementing data preprocessing strategies that account for ecological, technical, and methodological variability. Although the results from the models trained with a combined data set are not perfect, this should not detract from the potential of machine learning applications for seabed hydrocarbon seep microbiome data sets and predictions. Rather, this study points to a degree of microbiome uniqueness in different parts of the global ocean where hydrocarbon seepage is occurring, possibly owing to differences in petroleum geology or geochemistry as well as oceanography (36, 44). Nevertheless, consistency in the measurement of hydrocarbons, sediment sampling regimes, and the number of replicate sub-samples will all influence the effectiveness of machine learning models.

Future efforts, in this regard, should focus on standardizing hydrocarbon classification methods, such as developing models that distinguish between biogenic and thermogenic seeps rather than grouping all hydrocarbon sites into a hydrocarbon-positive category (45, 46). Furthermore, for finer-scale predictions, multinomial approaches could be considered to differentiate hydrocarbon types or to train on hydrocarbon-specific metrics such as C_1_/C_2+_ ratios, given that sufficient hydrocarbon samples are available and geochemical measurements are consistent between basins.

In addition, metabolic genes could be considered (i.e., focusing more directly on ecological functions) rather than the 16S rRNA gene-derived taxonomic data used here. Dedicated incorporation of archaea that may be diagnostic of hydrocarbon seepage was also not included in the analysis presented here. Less phylogenetic diversity typically characterizing relevant archaeal data sets (5) may result in models with less overfitting. It can also be considered whether training models on the entire amplicon library of samples may be excessive, and whether applying a higher relative abundance cut-off prevents models from overfitting to training data. Utilization of shotgun metagenomics data would allow greater mechanistic resolution to be obtained while reducing the noise from general community compositions (47). These approaches could also help identify ecological roles for important taxa such as *Campylobacterota* and *Caldatribacteriota*. Increasing the number of hydrocarbon-positive sampling sites, models could be developed to better capture true biological diversity using these other approaches. This, alongside ensuring consistent sampling depths and standardizing geochemical measurements across data sets would help mitigate overfitting and improve generalizability. With refined data sets and stronger controlled variables in sampling, machine learning models have the potential to be very effective tools in predicting ecological data, especially within a given basin or oceanic region.

## Data Availability

The Gulf of Mexico and NW Atlantic Scotian Slope 16S rRNA gene data sets used in this study are the basis of already published research in Chakraborty et al. (5) and Li et al. (6), respectively. The machine learning workflow utilized, and the specific code used to randomize the samples for the combined model testing is available on GitHub: https://github.com/rohan-ah-khan/16s-ASV-Metadata-ML-Prediction.

## SUPPLEMENTAL MATERIAL

Tables S1 and S2 spectrum.03033-24-s0001.xlsx The tables provide numeric data for the feature importance and AUC scores presented in the manuscript.

ASM does not own the copyrights to Supplemental Material that may be linked to, or accessed through, an article. The authors have granted ASM a non-exclusive, world-wide license to publish the Supplemental Material files. Please contact the corresponding author directly for reuse.
